# The Effectiveness of the Good Affordable Food Intervention for Adults with Low Socioeconomic Status and Small Incomes

**DOI:** 10.3390/ijerph17072535

**Published:** 2020-04-07

**Authors:** Kathelijne M.H.H. Bessems, Evelyne Linssen, Marion Lomme, Patricia Van Assema

**Affiliations:** 1 NUTRIM School of Nutrition and Translational Research in Metabolism, Maastricht University, P.O. Box 616, 6200MD Maastricht, The Netherlands; p.vanassema@maastrichtuniversity.nl; 2 Department of Knowledge & Innovation, Public Health Service South Limburg, P.O. Box 33, 6400 AA Heerlen, The Netherlands; Evelyne.Linssen@ggdzl.nl; 3 Dietician Practice Lomme, Lichtenberg 27, 6151BS Munstergeleen, The Netherlands; info@lomme.nl

**Keywords:** nutrition education, low socioeconomic status, nutrition literacy, procedural knowledge, determinants

## Abstract

Good Affordable Food (GAF) is a small-group nutrition education intervention for adults with low socioeconomic status and small incomes. It aims to empower participants to save money on groceries and consume healthier diets. This paper reports the short-term and longer-term effects on behavioural determinants and self-reported behavioural changes. A quasi-experimental control group design was applied with a baseline measurement, a post-test immediately after the intervention, and a follow-up measurement after six months. The study included 237 participants (intervention group: n = 131; control group: n = 106) at baseline, 197 at post-test, and 152 at follow-up. Data were collected by telephone, mostly using closed interview questions. Positive short-term and longer-term effects were found for attitude towards the costs of healthy foods, food label use, and the use of liquid butter or oil to prepare hot meals. Short-term intervention effects related to knowledge towards saving money on groceries, self-efficacy towards healthy eating, portion size awareness, and mindful eating. GAF was effective in changing some determinants and behaviours related to cost and food consumption, however, mostly in the short term. Thereby, it is an example of combining pricing and health information in nutrition education that developers of effective nutrition education for low-income groups can build on.

## 1. Introduction

The promotion of healthy diets is one of the key priorities for the prevention of chronic diseases, such as type 2 diabetes and cardiovascular disease [1]. As in many countries, most Dutch adults do not meet the national healthy food guidelines, particularly in relation to fruits, vegetables, sugar, and saturated fat [2,3]. In the Netherlands and other European countries, people of a lower socioeconomic status (SES), tend to consume unhealthier diets when compared to people of higher SES [4,5]. Unhealthy diets are often a result of a combination of unfortunate life situations that are concurrent with low SES, for example limited financial resources [6,7], increased psychological distress [8], and low health literacy [9,10,11]. Although many of these issues are intertwined and unlikely to be solved easily, health literacy is considered to be a changeable determinant that could be targeted to improve healthy food consumption [12] and, thereby, decrease socioeconomic disparities in health [13]. 

Health literacy has been defined as the knowledge, motivation, and competencies to access, understand, appraise, and apply health information in order to make judgements and take decisions in everyday life concerning health care, disease prevention, and health promotion to maintain or improve quality of life during the life course [14]. It comprises necessary reading and writing skills, skills to extract, derive meaning from and apply information in new situations, and more advanced skills to critically analyse information [15]. Different forms of health literacy have been recognized, including nutrition literacy [16,17]. Nutrition literacy has been distinguished into functional literacy and interactive or critical nutrition literacy. Functional literacy refers to “knowing what,” which relates to declarative knowledge, including reading and understanding information on factors that can affect health [16]. An example is knowing that frequent intake of products high in saturated fats is damaging for health [18]. Interactive or critical literacy refers to “knowing how” [16] and has been related to procedural knowledge, which is how to translate declarative knowledge into positive dietary change. For example, knowing how to replace products high in saturated fat with products low in fat to prepare a healthy meal would be procedural knowledge [18]. Making informed decisions about preparing healthy meals, requires substantial procedural knowledge [19], and it has been suggested to be particularly crucial for low-SES and low-health-literate groups [17,20,21,22].

Nutrition education interventions can contribute to improving the dietary behaviours of low-SES adults [23]. To bring about these changes, nutrition education interventions need to meet specific criteria. First, they should be comprehensive enough and target a number of determinants that interact with nutrition literacy, such as behavioural and environmental determinants [19,24,25]. Examples of behavioural determinants that have been found relevant and to be targeted in comprehensive interventions are misconceptions and awareness of personal consumption compared to recommendations [26], motivation or attitude [27], self-efficacy [28], taste and liking [29], food label use [30], portion size awareness [31], emotional and mindful eating [32], familiarity towards healthy foods [33,34], perceived convenience of preparation [35], planning [36], and (coping with negative) perceived social norms and lack of social support [28,37]. Environmental barriers for low-SES groups specifically, include the high price of healthy foods [38,39], limited financial resources and financial strain [40,41,42,43,44], and lower accessibility and availability [44]. Educational interventions should target how to deal with these environmental determinants that cannot be changed with nutrition education [20,23,24]. 

Second, educational interventions should use evidence-based behavioural change techniques for low-SES groups specifically [45,46,47]. The literature identifies some criteria for the application of behavioural change techniques to increase understanding, engagement, and memory among low-SES groups. For written and oral communication, the use of plain language is recommended, while technical language should be avoided [12,48,49]. Communication should be supported by photographs or relevant images, such as those depicting food products [12,49,50,51]. To check understanding, professionals should ask participants to explain instructions in their own words, also known as the teach-back method [52]. Finally, to actively engage participants, practical hands-on assignments are recommended that can be directly applied in the participants’ lives, such as interactive discussions [49,53] and demonstrations of simple uses of food labels [12].

Good Affordable Food (GAF) is a small-group healthy diet promotion intervention for low-SES adults. It aims to empower participants to increase their consumption of fruit and vegetables and decrease their consumption of saturated fat. Furthermore, the intervention aims to empower participants to save money on groceries. GAF was initiated in 2000 by a dietician and health promotion professionals from the Public Health Service South Limburg as a local response to socioeconomic health disparities and the underrepresentation of low-SES adults in health promotion programmes. South Limburg of the Netherlands is known for its high levels of low health literacy, high levels of poverty, and high prevalence rates of unhealthy lifestyles and dietary habits more specifically [54,55,56]. GAF was developed on request of the Limburg Credit Bank as a small local intervention as part of a household budgeting course for debt repayment clients of the bank, thereby reaching low-SES adults who were not reached through regular nutrition education interventions. Later, the programme was revised by a larger programme development team of two dieticians and health promotion experts of the Public Health Service and Maastricht University, and made available for general low-SES communities as well. GAF was initiated as part of poverty and health policies, but as the local context has changed over time, it was further developed to also fit social inclusion and health literacy policies of municipalities. An evaluation on dietary outcomes only of the first edition of the programme indicated effects in saturated fat intake and fruit juice consumption [57]. The aim of this study was to evaluate the present revised GAF intervention on behavioural determinants and self-reported changes in behaviour. The main research question was: “which short-term and longer-term effects does the GAF programme have on personal behavioural determinants and self-reported changes in dietary behaviours as well as on saving money when grocery shopping.”

## 2. Materials and Methods

### 2.1. The GAF Programme

The GAF programme comprises two 2-h sessions for a small group of 8–12 participants, with two weeks in between the sessions. The sessions are led by a trained course leader (dietician). GAF builds on theories on important elements of the behavioural change process, determinants of human behaviour, and self-management [58,59,60,61,62]. It acknowledges awareness of own behaviour as a first prerequisite for change, the importance of identifying and improving behavioural determinants that act as barriers for change, and personal goals and action plans as conditional for actual behavioural change. GAF focuses on a selection of the different determinants of dietary behaviour and saving money on groceries that were summarized earlier, including different types of knowledge, attitude, self-efficacy, familiarity with healthy foods, social support, and emotional and mindful eating. Learning objectives therefore relate to establishing awareness of the different targeted behaviours (e.g., knowing recommended intake of fruit, being aware of own intake of high-fat snacks, being aware of the discrepancy between own consumption of vegetables and recommended intake), improvement of the targeted behavioural determinants (e.g., thinking to be able to save money on groceries, having a positive attitude towards eating more healthily, having how-to knowledge on reading food labels), and action planning (making a plan for a small behavioural change, experiencing that small changes are feasible). The intervention builds upon the learning objectives that are intertwined and covered in the different assignments (Table 1). The mostly practical hands-on assignments in the small group allow the use of target group appropriate applications of behavioural change techniques for achieving the different learning objectives, such as active information processing, guided practice, new arguments, demonstration, goal setting, and food exposure.

### 2.2. Design

A quasi-experimental control group design was applied, with a baseline measurement 1–14 days before the first GAF session, a short-term post-test within two weeks after the second GAF session, and a follow-up 6 months after the second session. The intervention group (IG) comprised people living in South Limburg, who were either attending the two GAF sessions as part of their debt repayment trajectory (indicating that they had a living allowance below poverty level), or who participated in exactly the same GAF sessions organized in low-SES communities. All GAF sessions during the study were led by either one of the dieticians of the programme development team to assure high programme fidelity. The control group (CG) included low-income adults in the same region, who were either in a debt repayment trajectory or visited a local community centre for income-related support and who did not participate in any nutrition education intervention. 

#### Recruitment of Participants and Procedures

From 2012 until 2014, 237 participants gradually enrolled in the study. Data collection was completed 6 months after the second GAF session of the last enrolled participant. A research assistant recruited participants for the IG in person during one of the debt repayment trajectory meetings or by telephone after they subscribed for GAF sessions in their neighbourhoods. Likewise, participants for the CG were recruited in person during one of their debt repayment trajectory meetings or during their visit to one of three local community centres. Participants visited these local community centres to receive help from the public housing agency, the credit bank, or social welfare (indicators of a low income). Inclusion criteria for the study were being at least 18 years old, living in a low-SES neighbourhood in South Limburg, and a self-reported small income that leads to challenges paying fixed household costs. Overall, we intended to recruit a group of participants diverse in age, gender, educational level, origin, and work status and to synchronize the gradual enrolment of participants and related measurement times in the IG and CG. Professional interviewers were trained by the main researcher to collect the data for this study using a predefined interview script. After a pilot testing round, the scripts were finalized. All interviews were conducted by telephone. 

This study was part of a larger study on dietary consumption [64]. Each interview lasted about 45 min, with an average of 10–15 min for data collection for this study. As an incentive for study participation, study participants received gift vouchers of up to 50 euros. 

Before taking part in the study, participants signed an informed consent form. The Medical Ethical Committee of the Maastricht University Medical Centre+ considered this study not to be subject to the Medical Research Involving Human Subjects Act (WMO) according to Dutch standards, date 23 May 21012. The full protocol of the study was published at the International Standard Randomised Controlled Trial Number (ISRCTN) database. 

### 2.3. Measures

To limit research burden for the participants, a selection of salient outcomes targeted by the intervention was included. 

Background variables included age, gender, educational level, origin (country of birth of mother and father), and whether there was a household income from a paying job (from study participant and/or partner sharing the same home). These variables were only assessed at baseline in both the IG and CG. 

Nine determinants related to saving money on groceries and/or healthy food behaviours were assessed at baseline, post-test, and follow-up in both the IG and CG. The determinants were assessed with single items, either with five-point Likert scale values or dichotomous answering options. Perceived procedural knowledge was assessed with one item on how to save money (Do you know what you can do to save money on groceries? 1 = yes; 0 = no) and one item on healthy foods (Do you know what you can do to eat healthier? 1 = yes; 0 = no). One item assessed attitude towards the costs of healthy foods (Do you consider healthy foods costly? 2 = yes very expensive; −2 = no very cheap). Self-efficacy was assessed with one item towards saving money on groceries (Can you save money on your groceries if you want to? 2 = yes definitely; −2 = no definitely not), and an item towards eating healthier (Can you eat healthier if you want to? 2 = yes definitely; −2 = no definitely not). Finally, four items applied a similar five-point Likert scale (from 2 = yes always to −2 = no never) to assess reading food labels (Do you ever check food labels to know how much fat that product contains?), portion size awareness (Do you ever check the portion size, for the amount of the product you eat?), mindful eating (Are you ever unaware of what you are eating when you are doing something else, like watching television, driving, or working?), and emotional eating (Do you ever eat too much when you are feeling bored, tired, sad, or upset?).

Self-reported changes in saving money on groceries as a result of GAF, were assessed at post-test and follow-up in the IG only. These changes were assessed with two open-ended items (Were you able to save money on your groceries since <date of previous measurement>? What did you change?). Similarly, self-reported changes in dietary consumption were assessed (i.e., Did you improve your dietary consumption since <date of previous measurement>? What did you change?). The self-reported use of a cooking fat to prepare diner was assessed in the IG and CG with one item (Which type of fat product do you use to prepare the hot meal?) with four answering categories (butter, liquid butter, oil, other, namely).

### 2.4. Data Processing and Analysis

Educational level was categorized into three categories (low, intermediate, high) according to Dutch Standards [65]. Origin was defined as immigrant (i.e., non-western) if at least one of the parents had been born outside Europe (Turkey included as non-western) [63]. Household income from work was categorized into two categories (1 = study participant or partner living in the same house has an income from a paid job, 0 = none of the members of the household has a paid job). Attitude towards the costs of healthy foods, mindful eating, and emotional eating were recoded into the positive direction, indicating that a high score is a favourable score. The answers on the use of a cooking fat product were re-categorized into a favourable choice (liquid butter or oil) and an unfavourable choice (butter, comparable product high in saturated fat), in line with the Dutch healthy diet recommendations [63]. Open-ended answers on changes in dietary consumption and saving money on groceries were summarized and clustered into categories. Due to the small number of missing values, these were not imputed.

To test for selective dropout at post-test and follow-up, two logistic regressions were conducted with either the post-test or the follow-up score as the dependent variable, and condition, baseline score, age, gender, educational level, origin, household income as independent variables. To test for baseline differences between participants in the IG and CG, a logistic regression was conducted with condition as dependent variable and age, gender, educational level, origin, household income as independent variables. To assess intervention effects on each scale-measured determinant (e.g., attitude), two separate multiple linear regressions were conducted, one with the post-test score of the determinant as the dependent variable and condition, baseline score, age, gender, educational level, origin, household income as independent variables, and a similar regression analysis with follow-up score of the determinant as the dependent variable. Finally, to assess intervention effects on each dichotomously measured determinant (e.g., knowledge), two separate logistic linear regressions were conducted, with either the post-test or the follow-up score of the determinant as the dependent variable and condition, baseline score, age, gender, educational level, origin, household income as independent variables. All quantitative data were analysed in IBM SPSS Statistics 23 (SPSS Inc. Chicago, IL, USA).

## 3. Results

### 3.1. Response, Drop Out, and Population

The study included 237 participants (IG = 131, CG = 106) at baseline, 197 (83.1%) (IG = 108, CG = 89) at post-test, and 152 (64.1%) (IG = 80, CG = 72) at follow-up. At post-test, drop out was higher among younger participants (β = −0.062; *p* < 0.01) and males (males = 21.8%, females = 11.6%; OR = 0.319; *p* < 0.01). At follow-up, drop out was higher among younger participants (β = −0.029; *p* < 0.05) and participants with a household income from a paid job (with paid job income = 40.4%, without paid job income = 28.2%; OR = 0.524, *p* < 0.05). There was no significant difference in drop out between the IG and the CG. 

In total, 66.4% of the participants in the IG had participated in the budgeting course; the others had participated in the neighbourhood intervention (not shown in Tables). Background characteristics of the participants are shown in Table 2. Compared to the CG, the IG included significantly more males (IG: 42.6%; CG: 30.5%; *p* = 0.036). No other significant differences between the IG and the CG were found.

### 3.2. Effects on Behavioural Determinants and Self-Reported Changes in Targeted Behaviours

The IG reported more knowledge on how to save money at post-test but not at follow-up (Table 3). Furthermore, there was a beneficial short-term and longer-term effect on attitude towards the perceived costs of healthy foods. Regarding self-efficacy, there was no significant change towards saving money on groceries, but self-efficacy towards healthy eating changed in the short term. Finally, short-term favourable intervention effects were found for reading food labels, portion size awareness, and mindful eating. At follow-up, the effect on reading food labels persisted, but the effect on portion size and mindful eating was no longer significant. There was no effect on emotional eating in the short or longer term. Finally, there was a positive intervention effect on the use of liquid butter or oil in the short term and longer term.

At post-test, 56.5% (n = 61) of the 108 respondents from the IG reported that they had improved one or more dietary behaviours. At follow-up, 47.5% (n = 38) of the 80 respondents reported that they had started eating healthier since the post-test. The reported changes were very diverse, however. The most frequently mentioned changes were related to the category fruits and vegetables. Further, participants reported to have changed their consumption of products outside the Wheel of Five categories, improved their intake of nutrients such as fat and carbohydrates and more generic dietary habits such as changes in the frequency of meals, smaller portions, and variation in products (Table 4). 

At post-test, 41.6% (n = 45) of the 108 respondents reported that they had been able to save money on their groceries, at follow-up this was 37.5% (n = 30) of the 80 respondents. The most frequently mentioned changes were related to being more aware of promotions, expensive products, groceries, and awareness on the supermarket environment. A few participants mentioned changes related to wasting foods (Table 5). 

## 4. Discussion

The aim of this study was to assess the effects of the GAF intervention on determinants of dietary consumption and saving costs on groceries, and on self-reported changes in behaviours immediately after the intervention and after six months. The results reveal short-term positive effects on important determinants, including procedural knowledge and attitude regarding cost-related aspects of foods. Short-term effects were also found on other health-related determinants, including self-efficacy, food label use, portion size awareness, mindful eating, and the consumption of cooking fat to prepare hot meals. At follow-up however, only the effect on attitude towards saving costs on groceries, food label use, and the use of favourable types of cooking fat remained. No short-term and longer-term effects were found for self-efficacy towards saving money on groceries and emotional eating. The findings indicate that GAF has been effective in changing some determinants and behaviours related to cost and food consumption, though mostly in the short term.

Although GAF had a strong focus on increasing procedural knowledge, only a short-term effect was found on procedural knowledge towards saving money on groceries while no effects were found on procedural knowledge towards healthy eating. It is unclear whether this was due to lack of sensitivity of the single-item measures or due to the intervention itself. Others found that measurement issues are common in assessing nutrition knowledge and, therefore, call for validated measures [66]. 

The longer-term effects show that GAF was successful in achieving change in a combination of determinants. The longer-term effects on the use of food labels and attitude towards spending less money on healthy groceries are promising, as these relate to the important barriers, price [38] and limited food label use [30]. These findings are in line with the findings of evaluation studies of other nutrition education interventions in low-SES groups. However, these studies only assessed short-term changes in the use of food labels and knowledge and skills related to resource management [67,68]. Further, the effects on the consumption of liquid butter are in line with the evaluation study of the first version of the GAF intervention [57]. Overall, these findings can imply that we succeeded in applying the behavioural change techniques adequately to low-SES groups.

Still, the intervention effects are relatively small and not consistent for all outcomes, and most of the effects do not persist over time. It may not be realistic to expect consistent and large intervention effects on all outcomes due to the short intervention duration. The findings of our study support the findings of other research, which concluded that nutrition education interventions should be implemented as part of a comprehensive approach [23,69]. Nutrition education with a strong focus on increasing procedural knowledge could be combined with more skills-related cooking interventions that take into account planning and managing, selecting, preparing, and eating healthy foods [25,70,71]. Furthermore, nutrition education could be embedded in approaches that support creating supportive environments. By improved access and affordability of healthy foods and decreased price-related barriers, environments are created in which healthy choices are perceived as easier [23,72]. Examples of supporting environmental changes are vouchers and subsidies [73,74] and prizing interventions [75]. Altogether, these comprehensive approaches could potentially contribute to increased food literacy [25,76], healthier food behaviours [23], and in the long run to decreased socioeconomic disparities in health [1,11,77]. 

Our study had some strong points and limitations. A hard-to-reach relevant group participated in our study and we measured both direct and longer-term intervention effects. Because of the non-randomized evaluation design and the use of self-reported challenges with paying fixed household costs as indicators of a low income, our IG and CG were not fully comparable. As a partial solution for this issue, but also for the issue of selective dropout, we corrected for background characteristics in the regression analyses. Another weakness is that our measures were not validated and were based on self-reports, which is a method susceptible to recall bias and socially desirable answers. Finally, self-reported behavioural changes as a result of the programme were only assessed in the IG. Future research should evaluate comprehensive approaches, using validated measures that are adequate for low-SES groups, including the assessment of food literacy.

## 5. Conclusions

GAF moderately, but meaningfully, contributed to changing some behavioural determinants related to dietary consumption and money spent on groceries, as well as some behavioural changes. The GAF intervention is an example of how pricing and health information can be combined for low-income groups. Our study stresses the importance of targeting procedural knowledge in combination with other relevant determinants and applying evidence-based behavioural change techniques that are tailored to low-SES individuals. Finally, our study supports previously made statements that this type of nutrition education intervention is especially valuable as part of a comprehensive approach.

## Figures and Tables

**Table 1:** Overview of the assignments in the two sessions of the Good Affordable Food intervention.

Assignments (Materials)	
Session 1	Session 2
1. Welcome The course leader welcomes the participants and explains the setup of the course.	1. Welcome and look back The course leader welcomes the participants and explains the course schedule. This is followed by a plenary discussion on experiences with the action plans and the liquid butter. The course leader and the participants provide each other tips (worksheet for action plan).
2. Unexpected expenditures Participants discuss a scenario of someone who has to cut back on groceries due to an unexpected expenditure (worksheet with brief case description).	2. Rotation game The course leader explains the rotation game existing of 5 duo assignments as depicted below. The participants collaborate and support each other in conducting the assignments, and if needed, receive help from the course leader.
3. Prejudices and advantages Plenary brainstorm that is guided by a list of prejudices towards healthy eating, including countering of prejudices by sharing examples of affordable, tasty, and easy to prepare meals. To convince participants of the importance of healthy diets, the course leader draws a simple picture of a blood vessel with and without atherosclerosis (drawing of blood vessel).	2A. Snacks Based on the traffic-light information, participants choose snacks they consume regularly between meals, from the food categories cheese and meat products, savoury snacks, cookies, candy and chocolate, fried snacks, and ice cream. Participants are asked to replace a product from a higher-fat category (red) with a product from a lower-fat product from the same category or by fruit and vegetables (orange or green). They write down the price and saturated fat content of the replaced product (product wrappings with price and nutrient information labelled as red, orange, or green choice; worksheet for product label and price information).
4. A healthy daily menu The course leader shows a menu with the recommended daily amounts and portions of foods as proposed in the Wheel of Five information tool from the Netherlands Nutrition Centre [63]. Participants and the course leader share tips to meet the fruit and vegetable recommendations (real-life products and portions of recommended daily menu).	2B. Baking and frying The participants read food labels of baking products higher and lower in saturated fat and compare the prices of those products (product wrappings with price and nutrient information; worksheet for product label and price information).
5. Taste test activities Participants try out three different brands (and prices) of peanut butter and marmalade on bread and evaluate their tastes. This is followed by a discussion on misconceptions regarding the higher quality of A-brands (slices of sandwiches with topping, scoresheet to grade each product).	2C. Do not be seduced by your supermarket! A quiz in which participants examine pictures of food products and the supermarket surroundings to identify marketing techniques that trick people into buying products. They formulate tips on how to avoid those tricks (pictures of the inside of supermarket environment; worksheet for tricks and tips).
6. Your sources of saturated fat The course leader shows pictures of food product categories (i.e., cheese, meat products, meat, butter, dairy products, savoury snacks, cookies, candy and chocolate, fried snacks, and ice cream) with examples of high-fat products and lower-fat alternatives. Participants indicate from which categories (e.g., dairy products) they regularly consume high-fat products (e.g., whole milk) and which alternatives (e.g., skimmed milk) could replace them. This is followed by a discussion of a scenario involving emotional eating and a scenario involving unconscious eating. Participants reflect on their personal situations in which this occurs and give each other tips to deal with these situations (posters with pictures of products within red and orange product categories).	2D. Check the prices of your groceries Participants estimate the total price of four supermarket baskets containing 4–9 products each. All baskets cost around 5 euro but contain items from different brands and supermarkets demonstrating that conscious choices support saving money (food products of different brands and from different supermarket and 4 shopping baskets; worksheet for product and price information).
7. A plan for action Participants formulate their own personal action plan after a general discussion on how to deal with potential barriers. After one week, the course leader sends a text message reminder of the personal plan to each participant with a brief support message and reminder of the plan (worksheet for action plan, text message reminder).	2E. Fruit and vegetable quiz Quiz with questions and tips about fruits and vegetables (true or false quiz sheet).
8. Take home bottle of liquid butter After an explanation of how to use liquid butter, participants receive a bottle of liquid butter to try out at home (bottle of liquid butter).	3. Wrapping up A short evaluation of the course and participants receive low-fat cookies to try out at home (low-fat cookies).

**Table 2:** Background characteristics and differences between participants in the intervention group (IG) and control group (CG) at baseline (N = 237).

Background Characteristics	Mean (SD) or % CG (n = 106)	Mean (SD) or % IG (n = 131)	Odds Ratio (CI) Difference between IG and CG	*p*-Value
**Age**	44.3 (11.8)	44.5 (12.4)	0.992 (0.970–1.015)	0.500
**Gender**			0.544 (0.308–0.961)	0.036
Male	30.5	42.6		
Female	69.5	57.4		
**Educational level %**			0.888 (0.610–1.312)	0.550
Low	40.0	48.5		
Moderate	48.6	36.9		
High	11.4	14.6		
**Origin %**			0.611 (0.336–1.112)	0.107
Dutch	65.7	77.5		
Other	34.3	22.5		
**Income from paid job in the household %**			0.929 (0.529–1.632)	0.799
No paid job	63.2	64.6		
Paid job	36.8	35.4		

**Table 3:** Behavioural determinants and use of liquid butter or oil in the control group and intervention group at post-test (T1) and follow-up (T2): observed descriptive statistics and effects.

	Mean (SD) or Mean % CG			Mean (SD) or Mean % IG			T1: CG Versus IG (n = 197)		T2: CG Versus IG (n = 152)	
Behavioural Determinants	T0: (n = 106)	T1: (n = 89)	T2: (n = 72)	T0: (n = 131)	T1: (n = 108)	T2: (n = 80)	β or OR	*p*-Value	β or OR	*p*-Value
**Perceived procedural knowledge**										
Do you know how to save money on groceries? (yes/no)	80.2%	64.0%	70.8%	78.3%	88.0%	83.3%	5.463	0.000	2.266	0.072
Do you know what you can do to eat healthier? (yes/no)	79.3%	78.7%	81.9%	76.2%	86.1%	83.3%	2.265	0.063	1.469	0.432
**Attitude**										
Do you consider healthy foods to be costly? (−2 to 2)	−0.62 (0.72)	−0.56 (0.74)	−0.69 (0.78)	−0.65 (0.75)	−0.30 (0.73)	−0.42 (0.66)	0.207	0.001	0.218	0.004
**Self−efficacy**										
Do you think you could save money on your groceries if you wanted to? (−2 to 2)	0.72 (1.27)	0.56 (1.30)	0.76 (1.14)	0.64 (1.27)	0.75 (1.35)	0.78 (1.27)	0.102	0.142	0.016	0.844
Do you think you could eat healthier if you wanted to? (−2 to 2)	1.03 (1.03)	1.04 (1.02)	0.83 (1.13)	1.00 (0.95)	1.42 (0.83)	1.06 (1.02)	0.216	0.002	0.125	0.122
**Reading food labels**										
Do you ever check food labels to see how much fat products contain? (−2 to 2)	−0.34 (1.51)	−0.33 (1.48)	−0.33 (1.37)	−0.64 (1.46)	0.23 (1.35)	0.10 (1.42)	0.233	0.000	0.220	0.004
**Portion size awareness**										
Do you ever check the portion sizes of the products you eat? (−2 to 2)	−0.40 (1.46)	−0.33 (1.48)	−0.83 (1.25)	−0.40 (1.45)	−0.06 (1.55)	−0.23 (1.46)	0.167	0.015	−0.044	0.584
**Emotional eating**										
Do you ever eat too much when you are feeling bored, tired, sad or upset? (−2 to 2)	0.69 (1.10)	0.88 (1.16)	0.88 (0.96)	0.75 (1.06)	1.10 (1.05)	1.15 (0.97)	0.112	0.087	0.123	0.081
**Mindful eating**										
Are you ever unaware of what you are eating when you are doing something else, like watching television, driving or work? (−2 to 2)	0.97 (1.11)	−0.52 (1.31)	0.96 (1.08)	0.97 (1.09)	1.28 (1.00)	1.04 (1.17)	0.126	0.050	0.084	0.288
**Type of fat product to prepare hot meal**										
Favourable product (0/1)	36.8%	32.6%	36.1%	33.9%	56.9%	52.6%	3.991	0.001	2.953	0.014

Notes: All linear and logistic regressions included condition, baseline score, age, gender, educational level, origin, household income as independent variables.

**Table 4:** Self-reported changes in dietary behaviours in the intervention group at post-test (T1; n = 108) and follow-up (T2; n = 80).

Self-Reported Changes in Dietary Behaviours	T1 (n = 61)	T2 (n = 38)
**Fruit and vegetables**	34	17
More fruit	22	7
More vegetables	9	10
More fruit juice	2	-
More vegetable juice	1	-
**Fish, legumes, eggs, nuts, and dairy products**	15	10
More fish	5	2
Fewer high-fat dairy products (e.g., milk)	4	2
More lower-fat dairy products (e.g., cheese)	3	-
More dairy products	2	1
Less meat (products) or replace meat by legumes	1	5
**Butter and cooking fats**	10	9
Liquid butter or oil to prepare hot meal	6	4
Low-fat margarine on bread	3	2
Less butter to prepare hot meal	1	3
**Bread, grains, and potatoes**	5	1
More wholegrain bread	3	1
More bread	2	-
**Drinks**	9	6
More water	5	3
Fewer soft drinks and/or energy drinks	4	3
**Other products than products from Wheel of Five categories**	16	8
Fewer fried snacks and fries	7	1
Less candy	5	5
Lower-fat sandwich spreads	2	1
More lower-fat candy and cookies	1	-
Light products	1	1
**Change in nutrients**	13	7
Reducing fat intake	6	4
Reducing intake of carbohydrates	3	2
Reducing intake of sodium	2	1
Increasing fibre intake	2	-
**Food-related behaviours**	17	16
Increased awareness or mindfulness of own intake	6	3
Reading food labels	3	1
Smaller portions	2	-
Consume breakfast	2	1
Overall healthier diet	1	4
More variation in meals and products	1	3
Eating more (in case of underweight)	1	-
Consume regular meals	1	2
Preparing own meal	-	2
**Total**	117	65

**Table 5:** Self-reported changes in saving money on groceries in the intervention group at post-test (T1; n = 108) and follow-up (T2; n = 80).

Self-Reported Changes in Saving Money on Groceries	T1 (n = 45)	T2 (n = 30)
**Awareness**	55	30
Promotions	17	9
Brand awareness, not buying costly products (e.g., meat)	17	14
Thrifty, more aware of how to spend money	12	6
Being more aware of supermarket environment (place of cheaper products, comparing prices, shopping list)	9	1
**Groceries**	12	5
Grocery shopping in other shops or at the market	5	1
Grocery shopping in more shops comparing prices	2	2
Frequency of grocery shopping	4	2
Grocery shopping after meal (to limit feelings of hunger)	1	-
**Other**	6	9
Not wasting foods, freezing meals and products	2	4
Preparing less expensive meals	1	-
Applying for support from the food bank	1	-
Having dinner at a family member’s home	1	1
Growing a kitchen garden	-	2
Quit smoking	1	-
Buying seasonal products	-	2
**Total**	73	44
